# Intersite reproducibility of myocardial assessment of T1 and partition coefficient on phantom and one volunteer at 1.5T and 3.0T

**DOI:** 10.1186/1532-429X-16-S1-P79

**Published:** 2014-01-16

**Authors:** Claire Falque, Frank Kober, Monique Bernard, Alexis Jacquier

**Affiliations:** 1 Radiology, APHM, Marseille, France; 2 CRMBM, Aix Marseille Université UMR CNRS n° 7339, Marseille, France

## Background

Myocardial extracellular volume is proportionate to partition coefficient (λ). λ can be measured by assessing the T1 relaxation time before and after injection of gadolinium. T1 and λ might become clinically relevant parameters for prognostic assessment. The goal of that study is to assess the intersite reproducibility of T1 assessment and partition coefficient on phantom and on one volunteer at 1.5 and 3.0T using ShMOLLI sequences.

## Methods

Twelve national institutions agreed to be included in the study (eleven 1.5T magnets and two 3.0T magnets). All of the magnets were different models of Siemens manufacturer (Erlangen, Germany). Phantoms were built to produce similar T1 and T2 values of myocardium and blood before and after gadolinium administration. Phantoms T1 reference values were assessed ones using TSE(TR/TE = 13000/18; 17 inversions between 30-9000 ms, turbo = 7). 2 ShMOLLI schemes were tested: for precontrast T1, we used scheme with 2 inversion (5 and 2 sampling scheme). For post contrast T1, we employed 3 inversion pulses with 4, 3, 2 scheme. At 1.5T, we used a pause of 3 heartbeats to recover before the next inversion pulse and α = 35°. For 3.0T acquisitions, we used similar schemes but with α = 20° and a pause of 4 heartbeats to recover for precontrast acquisition. On all sites, phantoms T1 mapping were assessed with an ECG simulation at heart rate 40, 60, 90 and 120. λ value were simulated using phantom matching with pre and post contrast with the following equations: R1 = 1/T1 ΔR1 = (R1postcontrast-R1precontrast) ΔR1ratio=λ=ΔR1myocardium/ΔR1blood For the volunteer study, same ShMOLLI schemes were performed on one volunteer on 7 magnets (five 1.5T and two 3.0T) for blood, fat, skeletal muscle and myocardial T1 quantification without contrast injection.

## Results

**Figure 1:**
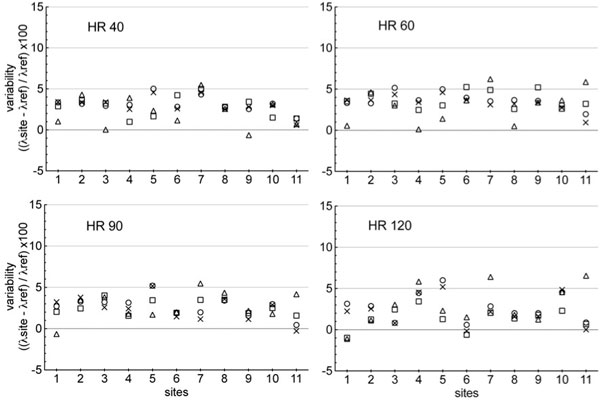
**Graph showing the partition coefficient (λ) calculated for all the 1.5T centers in comparison to those of reference at each heart rate tested, with △ corresponding to normal myocardium, ■ corresponding to myocardium with low level of fibrosis, × corresponding to myocardium with moderate level of fibrosis, and ○ to myocardium with high level of fibrosis**.

**Figure 2:**
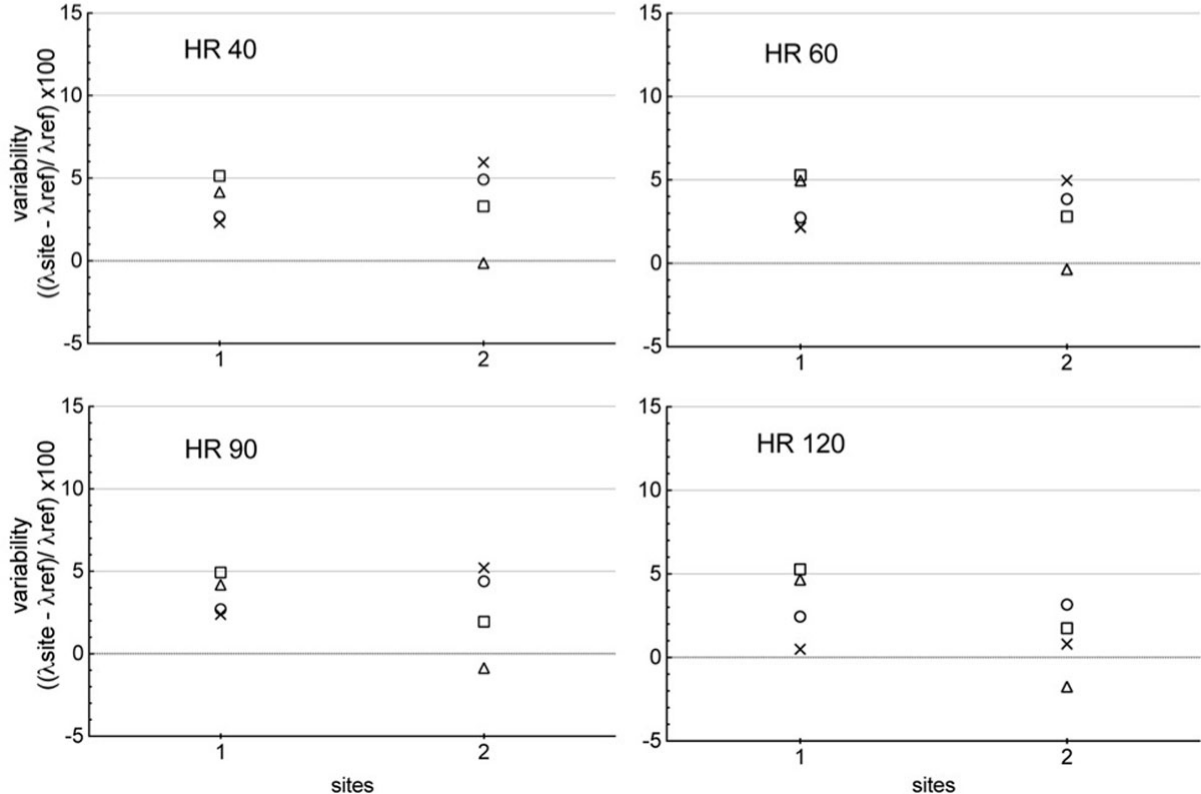
**Graph showing the partition coefficient (λ) calculated for all the 3.0T centers in comparison to those of reference at each heart rate tested, with △ corresponding to normal myocardium, ■ corresponding to myocardium with low level of fibrosis, × corresponding to myocardium with moderate level of fibrosis, and ○ to myocardium with high level of fibrosis**.

For the phantom analysis, at 1.5T (11 magnets), partition coefficient was over estimated by 2.9% (1.8; 3.4) at 40 bpm, 3.4%(3.0; 4.2) at 60 bpm, 2.0%(1.7; 3.5) at 90 bpm and 2.0% (1.2; 4.4) at 120 bpm (Figure 1). At 3.0T, partition coefficient was over estimated by 3.7% (2.4; 5.0) at 40 bpm; 3.3% (2.3; 4.9) at 60 bpm; 3.4% (2.0; 4.8) at 90 bpm; 2.0% (0.5; 4.2) at 120 bpm (Figure 2). For the volunteer, the mean T1 value for fat, myocardium, skeletal muscle and blood was: 306.1 ± 19.1 ms; 947.2 ± 20.4 ms; 796.2 ± 20.5 ms; 1476.0 ± 27.6 ms respectively at 1.5T, and 383.0 ± 11.5 ms; 1176.0 ± 21.6 ms; 1072.0 ± 9.8 ms; 1749.0 ± 39.7 ms at 3.0T. Concerning T1 values obtained in myocardium, blood, fat and skeletal muscle there were a good reproducibility between the different sites at 1.5T and 3T.

## Conclusions

Lambda estimation is precise and reproducible between different sites at 1.5T and 3.0T using ShMOLLI. For the volunteer, the T1 values obtained in the different sites were similar.

## Funding

This study was supported by the PHRC 2011-A00887 - 34.

